# The effect of exogenous glucagon on circulating amino acids in individuals with and without type 2 diabetes and obesity

**DOI:** 10.1530/EC-23-0516

**Published:** 2024-02-21

**Authors:** Magnus F G Grøndahl, Jonatan I Bagger, Malte P Suppli, Gerrit Van Hall, Nicolai J W Albrechtsen, Jens J Holst, Tina Vilsbøll, Mikkel B Christensen, Asger B Lund, Filip K Knop

**Affiliations:** 1 Center for Clinical Metabolic Research, Gentofte Hospital, University of Copenhagen, Hellerup, Denmark; 2 Clinical Research, Steno Diabetes Center Copenhagen, Herlev, Denmark; 3 Department of Clinical Biochemistry, Clinical Metabolomics Core Facility, Rigshospitalet, University of Copenhagen, Copenhagen, Denmark; 4 Department of Biomedical Sciences, Faculty of Health and Medical Sciences, University of Copenhagen, Copenhagen, Denmark; 5 Department of Clinical Biochemistry, University Hospital Copenhagen, Bispebjerg, Copenhagen, Denmark; 6 Novo Nordisk Foundation Center for Basic Metabolic Research, Faculty of Health and Medical Sciences, University of Copenhagen, Copenhagen, Denmark; 7 Department of Clinical Medicine, Faculty of Health and Medical Sciences, University of Copenhagen, Copenhagen, Denmark; 8 Department of Clinical Pharmacology, Copenhagen University Hospital – Bispebjerg and Frederiksberg, Copenhagen, Denmark; 9 Copenhagen Center for Translational Research, Copenhagen University Hospital – Bispebjerg and Frederiksberg, University of Copenhagen, Copenhagen, Denmark

**Keywords:** amino acids, glucagon, obesity, type 2 diabetes

## Abstract

**Objective:**

In obesity and type 2 diabetes, hyperglucagonaemia may be caused by elevated levels of glucagonotropic amino acids due to hepatic glucagon resistance at the level of amino acid turnover. Here, we investigated the effect of exogenous glucagon on circulating amino acids in obese and non-obese individuals with and without type 2 diabetes.

**Design:**

This was a *post hoc* analysis in a glucagon infusion study performed in individuals with type 2 diabetes (*n* = 16) and in age, sex, and body mass index-matched control individuals without diabetes (*n* = 16). Each group comprised two subgroups of eight individuals with and without obesity, respectively.

**Methods:**

All participants received a 1-h glucagon infusion (4 ng/kg/min) in the overnight fasted state. Plasma amino acid concentrations were measured with frequent intervals.

**Results:**

Compared to the control subgroup without obesity, baseline total amino acid levels were elevated in the control subgroup with obesity and in the type 2 diabetes subgroup without obesity. In all subgroups, amino acid levels decreased by up to 20% in response to glucagon infusion, which resulted in high physiological steady-state glucagon levels (mean concentration: 74 pmol/L, 95% CI [68;79] pmol/L). Following correction for multiple testing, no intergroup differences in changes in amino acid levels reached significance.

**Conclusion:**

Obesity and type 2 diabetes status was associated with elevated fasting levels of total amino acids. The glucagon infusion decreased circulating amino acid levels similarly in all subgroups, without significant differences in the response to exogenous glucagon between individuals with and without obesity and type 2 diabetes.

**Significance statement:**

The hormone glucagon stimulates glucose production from the liver, which may promote hyperglycaemia if glucagon levels are abnormally elevated, as is often seen in type 2 diabetes and obesity. Glucagon levels are closely linked to, and influenced by, the levels of circulating amino acids. To further investigate this link, we measured amino acid levels in individuals with and without obesity and type 2 diabetes before and during an infusion of glucagon. We found that circulating amino acid levels were higher in type 2 diabetes and obesity, and that glucagon infusion decreased amino acid levels in both individuals with and without type 2 diabetes and obesity. The study adds novel information to the link between circulating levels of glucagon and amino acids.

## Introduction

The peptide hormone glucagon increases hepatic glucose production and is secreted from the pancreatic alpha cells when glucose mobilisation via gluconeogenesis or glycogenolysis is needed, e.g. during hypoglycaemia (1). Besides glucagon’s well-known effects on glucose metabolism, the hormone is also involved in amino acid metabolism. Glucagon secretion is stimulated by glucagonotropic amino acids and glucagon in turn stimulates hepatic amino acid breakdown (2). Elevated levels of glucagon, hyperglucagonaemia, are observed in several obesity-related diseases including type 2 diabetes and non-alcoholic fatty liver disease where it contributes to an increased risk of hyperglycaemia (3, 4, 5, 6, 7, 8, 9). The mechanisms underlying this hyperglucagonaemia remain unclear, but may include elevated circulating levels of glucagonotropic amino acids as a consequence of an impairment of amino acid turnover, perhaps as a result of resistance towards this action of glucagon (10, 11, 12, 13); possibly explaining why hyperglucagonaemia and hyperaminoacidaemia often go hand in hand in these conditions. This impairment is thought to arise due to hepatic steatosis, of which obesity is a primary risk factor (7, 14).

To better understand the relationship between circulating amino acids and glucagon in obesity and type 2 diabetes, we evaluated circulating amino acid levels in individuals with and without obesity and with and without type 2 diabetes before and in response to a 1-h intravenous glucagon infusion.

## Materials and methods

### Study design and participants

Plasma samples from a study including 16 individuals with diet and/or metformin-treated type 2 diabetes (type 2 diabetes group) and 16 individuals without diabetes (control group) conducted at our facility were analysed (15). The study was approved by the Scientific-Ethical Committee of the Capital Region of Denmark (registration no. H-1-2014-066) and registered with ClinicalTrials.gov (ID: NCT02475421). The study was performed in accordance with the principles of the Declaration of Helsinki (Seventh Revision, 2013), and consent was obtained from each participant after a full explanation of the purpose and nature of all procedures used. The two main groups were carefully matched on age, sex, and BMI and differed significantly only on HbA1c and Homeostatic Model Assessment for Insulin Resistance (HOMA-IR). The two main groups were intentionally recruited to provide two subgroups (*n* = 8 in each), with either a BMI below 27 kg/m^2^ (subgroups without obesity) or above 33 kg/m^2^ (subgroups with obesity). All participants had normal kidney and liver function.

### Procedures and analyses

All participants completed one experimental day after an overnight fast, including liquids, tobacco, and medication. Any metformin treatment was paused 7 days before the experimental day. Blood samples were frequently drawn before, during, and after a 1-h intravenous infusion of glucagon (4 ng/kg/min, GlucaGen, Novo Nordisk A/S). Amino acid levels were measured in plasma from blood drawn into chilled tubes containing EDTA, aprotinin, and valine pyrrolidine. Liver fat content was evaluated by measuring the controlled attenuation parameter (CAP) using transient elastography (FibroScan Touch 502, Echosens SA, France) (16). Amino acids were quantified by liquid chromatography–tandem mass spectroscopy (LC-MS/MS Quantiva, Thermo Scientific) (17).

### Statistical analyses

The amino acid data reported in this article were analysed *post hoc*. The original sample size calculation has been published previously and was based on the detection of physiologically relevant changes in metabolic clearance rate of glucagon between individuals with and without type 2 diabetes and obesity, respectively (15). The effect of the glucagon infusion on amino acid levels was calculated as the difference between the mean value of two amino acid measurements 30 and 15 min before the start of infusion (baseline) and the mean value of two amino acid measurements 55 and 60 min after the start of infusion, during glucagon steady-state conditions (15). Change from baseline is presented as both absolute and relative change. Skewed data were log-normalised before analysis. Student’s *t*-test was used to compare the two main groups and the four subgroups. Correction for multiple testing was done using the false discovery approach by Benjamini and Hochberg (18). Adjustments for differences in insulin sensitivity between groups were made using a linear mixed model. Both the results of raw and adjusted *P*-values are reported. A two-sided *P <* 0.05 was chosen to indicate statistical significance. Data are reported as mean with 95% confidence intervals (CI) in brackets or standard deviation (s.d.) in parentheses unless otherwise stated. Data were handled using GraphPad Prism v9.4.1, Microsoft Excel, and R version 4.1.2.

## Results

Results of the original study are published elsewhere (15). In summary, fasting glucagon levels were lower in the control subgroup without obesity compared to the other three subgroups. Glucagon levels rose in all participants following glucagon infusion, reaching steady-state levels at high physiological levels (mean concentration of 74 pmol/L; 95% CI [68;79] pmol/L) after 40 min of infusion. Mean steady-state plasma glucagon concentrations were slightly higher in the control group compared to the type 2 diabetes group, primarily due to the control subgroup with obesity having higher steady-state concentrations than the other three subgroups. Fasting levels of insulin were expectedly elevated in the type 2 diabetes group compared to the control group. The control subgroup without obesity had significantly lower fasting insulin levels than the control subgroup with obesity. In response to the glucagon infusion, the control group exhibited a larger increment in insulin levels compared to the type 2 diabetes group (15).

### Transient elastography

No differences in CAP values were detected between the type 2 diabetes group (293 ± 49 dB/m) and the control group (279 ± 69 dB/m), while the control subgroup without obesity (241 ± 43 dB/m) had significantly lower CAP values compared to both the control subgroup with obesity (321 ± 70 dB/m, *P* = 0.03) and the type 2 diabetes subgroup with obesity (307 ± 39 dB/m, *P* = 0.01).

## Amino acid concentrations

### Fasting amino acids

**Figure 1:**
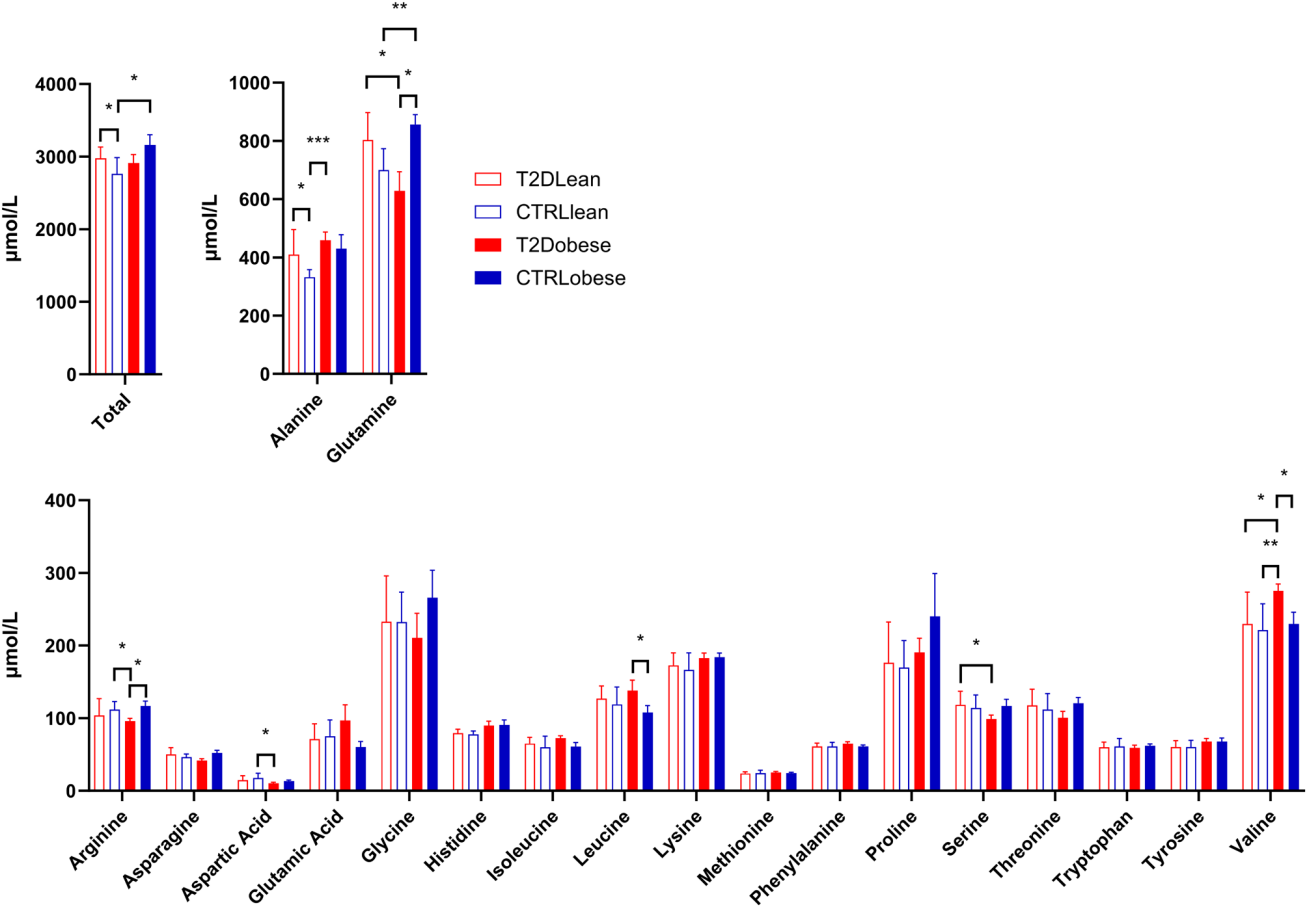
Fasting baseline levels of amino acids. Data are mean ± s.d. Open bars: subgroups without obesity; solid bars: subgroups with obesity; red bars: subgroups with type 2 diabetes; blue bars: subgroups without type 2 diabetes. Asterisks denote uncorrected statistical significance (*P < 0.05, **P < 0.01, and ***P < 0.001, respectively). T2D, type 2 diabetes.

Total fasting amino acid concentrations are reported in Table 1 and Fig. 1. Individual fasting amino acid concentrations are reported in Supplementary Table 1 (see section on supplementary materials given at the end of this article) and Fig. 1. Compared to the control subgroup without obesity, baseline total amino acid levels were elevated in the control subgroup with obesity and in the type 2 diabetes subgroup without obesity. Furthermore, baseline differences between subgroups were observed for glutamine, alanine, valine, leucine, serine, arginine, and aspartic acid. Following correction for multiple testing, no differences remained significant.

**Table 1:** Total amino acid levels.

	Type 2 diabetes group	Control group
**Baseline amino acid levels (µmol/L)**		
Main group (*n* = 16)	2942 [2816–3068]	2961 [2778–3144]
Subgroup without obesity (*n* = 8)	2977 [2870–3084]^a^	2763 [2609–2917]^a,b^
Subgroup with obesity (*n* = 8)	2908 [2673–3143]	3160 [2883–3437]^b^
**Absolute change from baseline (µmol/L)**		
Main group (*n* = 16)	−246 [−305–−187]	−272 [−323–−221]
Subgroup without obesity (*n* = 8)	−285 [−365–−205]	−268 [−340–−196]
Subgroup with obesity (*n* = 8)	−206 [−289–−123]	−275 [−353–−197]
**Relative change from baseline (%)**		
Main group (*n* = 16)	−8.4 [−10.5–−6.3]	−9.3 [−11.1–−7.5]
Subgroup without obesity (*n* = 8)	−9.7 [−12.7–−6.7]	−9.7 [−12.3–−7.1]
Subgroup with obesity (*n* = 8)	−7.2 [−10.1–−4.3]	−8.8 [−11.5–−6.1]

Baseline values and responses to glucagon infusion in total amino acid levels. Right column: individuals without diabetes (control group). Middle column: individuals with type 2 diabetes (type 2 diabetes group). Each group is displayed on the main group level (*n* = 16) and subgroup levels (*n* = 8), respectively. Data are presented as means with 95% CIs in brackets.

^a^Denotes raw statistical significance (*P* < 0.05) between the type 2 diabetes group and the control group; ^b^Denotes raw statistical significance (*P* < 0.05) between the lean and obese subgroups.

### Amino acid levels in response to exogenous glucagon infusion

**Figure 2:**
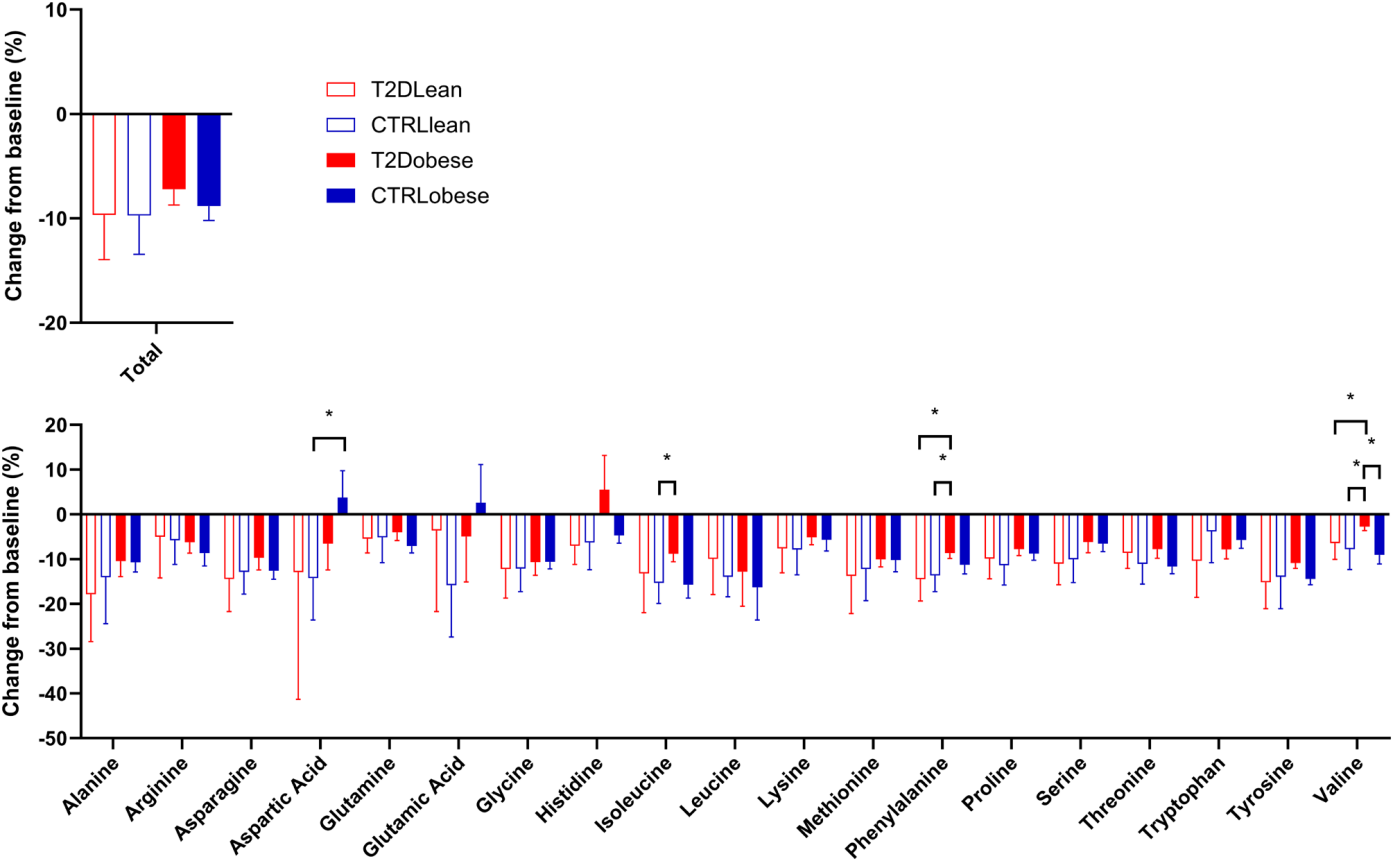
Relative change from baseline in amino acid levels in response to the glucagon infusion. Data are mean ± s.d. Open bars: lean subgroups; solid bars: obese subgroups; red bars: participants with type 2 diabetes; blue bars: participants without type 2 diabetes. Asterisks denote uncorrected statistical significance (*P < 0.05). T2D, type 2 diabetes.

Change from baseline in total amino acids is reported in Table 1 and Fig. 2. Change from baseline in individual amino acids is reported in Supplementary Table 3 and Fig. 2. Total amino acids decreased in all participants with a mean relative change of −8.9 [−10.2–−7.5] %. We observed no significant differences between the two main groups in the change in total amino acid levels following glucagon infusion. Concentrations of nearly every individual amino acid decreased during the glucagon infusion, with the largest decreases nearing 15–20% for amino acids such as alanine, isoleucine, and leucine (Fig. 2). Differences between subgroups in relative change from baseline were observed for some individual amino acids such as a larger decrease in aspartic acid levels in the control subgroup without obesity compared to the control subgroup with obesity and smaller decreases in the type 2 diabetes subgroup with obesity for valine, phenylalanine, and isoleucine; however, following correction for multiple testing, these differences were not statistically significant. In a linear mixed model, we found no significant effect of either fasting insulin (*P = *0.21), insulin response (*P = *0.80), and HOMA-IR (*P = *0.38) on change from baseline in amino acid levels.

## Discussion

Here, we investigated circulating amino acid levels in participants with and without obesity and type 2 diabetes in both the fasting state and during an intravenous glucagon infusion (dosed to achieve high physiological plasma concentrations). In the control subgroup without obesity, who were expected to be the most metabolically healthy of the four subgroups, we observed the lowest fasting concentrations of total amino acids, glucagon, insulin, and hepatic fat content (as measured by CAP). This corroborates the theory of a link between hepatic steatosis, glucagon concentrations, and amino acid levels and fits well with the liver–alpha cell axis concept. Interestingly, we observed lower fasting concentrations of the glucagonotropic amino acid alanine in the control subgroup without obesity (Fig. 2). Alanine has been proposed as a key amino acid in the liver–alpha cell feedback loop, and elevated alanine levels could be a sign of an increased hepatic glucagon resistance (13, 19). Importantly, prevailing circulating concentrations of insulin could also affect the alanine levels observed, as these are intrinsically linked as well (20). Of note, we did not find significantly elevated levels of total fasting amino acids in the type 2 diabetes subgroup with obesity, who were expected to be the most metabolically unhealthy subgroup, with the highest risk of hyperaminoacidaemia. This may be due to the severely elevated fasting insulin levels reflecting the overall increased insulin resistance in the subgroup. Insulin is a potent regulator of whole-body amino acid metabolism (21, 22), and the difference in both fasting insulin levels and the different insulin response to the glucagon infusion makes it difficult to evaluate the isolated effect of glucagon on amino acid levels. The exogenous glucagon infusion decreased total amino acid levels significantly in all groups. In the subgroups with obesity, we observed trends towards smaller responses to the glucagon infusion regarding some amino acids such as aspartic acid and valine, but overall, the effect of glucagon was similar in all groups with no clear effects of type 2 diabetes or obesity on the response to the high physiological glucagon levels. Thus, our results may suggest that high levels of glucagon can overcome the proposed hepatic glucagon resistance on amino acid metabolism. Notably, this study cannot isolate the effect of the liver on circulating amino acid levels as extrahepatic mechanisms (e.g. muscle protein metabolism and renal amino acid handling for both gluconeogenesis and nitrogen excretion) also play a role in the amino acid changes observed following glucagon infusion. This is evident from the differing response in insulin secretion following glucagon infusion. However, when evaluating the potential interference from the differing insulin levels, we found no correlation between either insulin levels or HOMA-IR and the effect of glucagon on amino acid levels. Preferably, a pancreatic clamp should have been performed, eliminating influence from endogenous insulin secretion. Furthermore, from this study, we cannot elucidate potential hidden changes in amino acid metabolism as this would have required the use of tracer techniques.

The study evaluated participants who were similar in sex and age, with a large intentional difference in the lean and obese subgroups regarding BMI, and a fairly long mean duration of type 2 diabetes of 7.5 years, thus providing a sound basis for evaluating the effect of both obesity and type 2 diabetes on changes in plasma amino acid concentrations in response to exogenous glucagon (15). The Caucasian ethnicity and narrow age group (mean age 59 years; 95% CI [56;62] years), nevertheless, limit the generalisability of the data. Also, the limited sample size, the multiple amino acids measured; thus, the multiple comparisons combined with the *post hoc* nature of the analyses limit the power of our statistical comparisons between groups. Furthermore, the correction for multiple testing increases the risk of type 2 errors. Data should be interpreted with these limitations in mind.

In this *post hoc* study, we find that type 2 diabetes and obesity are associated with increased fasting amino acid levels, and that an intravenous glucagon infusion of 4 ng/kg/min acutely lowers circulating amino acid concentrations in both individuals with and without type 2 diabetes and with and without obesity. The lack of differences between groups with/without obesity/type 2 diabetes needs to be considered in the light of our limited sample size, multiple comparisons and differences in prevailing circulating concentrations of insulin.

## Supplementary Materials

Supplemental Material

## Declaration of interest

MFGG has received lecture fees from Novo Nordisk and is a minority shareholder of Zealand Pharma. JIB has received lecture fees from Novo Nordisk. JJH was on the advisory panel of, was a consultant for, was in the speaker’s bureau of, and/or has received research support from AstraZeneca, GlaxoSmithKline, Hamni, Intarcia, Merck Sharp & Dohme, Novartis, Novo Nordisk, Sanofi, and Zealand Pharma. TV is on the advisory panel for, is a consultant for, is in the speaker’s bureau of, and/or has received research support from AstraZeneca, Boehringer Ingelheim, Bristol-Myers Squibb, Eli Lilly, Gilead, GSK, Merck Sharp & Dohme, Novartis, Novo Nordisk, Sanofi, and Sun Pharmaceuticals. FKK is on the advisory panel of, is a consultant for, is in the speaker's bureau of, owns shares in, and/or has received research support from 89bio, AstraZeneca, Boehringer Ingelheim, Cytoki Pharma, Eli Lilly, Gubra, Novo Nordisk, Merck Sharp & Dohme, Sanofi, Structure Therapeutics, Zealand Pharma, and Zucara; and is co-founder of and a minority shareholder in Antag Therapeutics. ABL, GvH, MBC, MPS, and NJWA have nothing to declare.

## Funding

The present work was funded by the Novo Nordiskhttp://dx.doi.org/10.13039/501100004191 Foundation. The funding party has not been involved in the design, data collection, data analysis, data interpretation, writing, nor in the publication of the study.

## Data availability

The datasets from the current study are available from the corresponding author upon reasonable request.

## Author contribution statement

MFGG and ABL planned and conducted the clinical experiments. MFGG researched data, performed statistical analyses, and wrote the manuscript. JIB, MBC, TV, JJH, and NJWA planned the study. MPS researched data. GvH provided the analyses of amino acids. FKK conceptualised the study, wrote applications for funding, planned the study, and wrote the manuscript. All authors contributed to the discussion and critically reviewed the manuscript. MFGG and FKK are the guarantors of this work and, as such, had full access to all the data.
