# Supplementary material for: The effect of exogenous glucagon on circulating amino acids in individuals with and without type 2 diabetes and obesity
Source: Endocr Connect. 2024 Feb 21;13(3):e230516. doi: 10.1530/EC-23-0516 (PMC10959036; doi:10.1530/EC-23-0516)
Supplement: Supplemental Material [file supplementary_material.pdf]

The Effect of Exogenous Glucagon on Circulating Amino Acids in Individuals with and without Type 2 Diabetes and Obesity

Supplemental material

Magnus F. G. Grøndahl[1], Jonatan I. Bagger[1,2], Malte P. Suppli[1], Gerrit Van Hall[3,4], Nicolai J. W. Albrechtsen[6], Jens J. Holst[4,5], Tina Vilsbøll[1,2], Mikkel B. Christensen[1,7,8,9], Asger B. Lund[1] and Filip K. Knop*[1,2,5]

[1]Center for Clinical Metabolic Research, Gentofte Hospital, University of Copenhagen, Hellerup, Denmark; [2]Clinical Research, Steno Diabetes Center Copenhagen, Herlev, Denmark; [3]Clinical Metabolomics Core Facility, Department of Clinical Biochemistry, Rigshospitalet, University of Copenhagen, Copenhagen, Denmark; [4]Department of Biomedical Sciences, Faculty of Health and Medical Sciences, University of Copenhagen, Copenhagen, Denmark; [5]Novo Nordisk Foundation Center for Basic Metabolic Research, Faculty of Health and Medical Sciences, University of Copenhagen, Copenhagen, Denmark; [6]Department of Clinical Biochemistry, University Hospital Copenhagen, Bispebjerg, Copenhagen, Denmark, [7]Department of Clinical Medicine, Faculty of Health and Medical Sciences, University of Copenhagen, Copenhagen, Denmark; [8]Department of Clinical Pharmacology, Copenhagen University Hospital - Bispebjerg and Frederiksberg, Copenhagen, Denmark; [9]Copenhagen Center for Translational Research, Copenhagen University Hospital - Bispebjerg and Frederiksberg, University of Copenhagen, Copenhagen, Denmark;

*Correspondence: Professor Filip Krag Knop, MD PhD, Director of Center for Clinical Metabolic Research, Gentofte Hospital, University of Copenhagen, Gentofte Hospitalsvej 7, 3rd floor, DK-2900 Hellerup, Denmark; tel.: +45 38674266; fax: +45 38677661; email: filip.krag.knop.01@regionh.dk; ORCID number 0000-0002-2495-5034

Key words: Glucagon, amino acids, obesity, type 2 diabetes

Supplemental material

Table S1

	Type 2 diabetes group	Control group
Alanine		
Main group (n=16)	435 [393;476]	381 [329;434]
Subgroup without obesity (n=8)	410 [351;469]	332 [314;350]
Subgroup with obesity (n=8)	459 [404;515]	430 [336;524]
Arginine		
Main group (n=16)	99.8 [91.0;109]	114 [107;122]
Subgroup without obesity (n=8)	104 [87.7;120]	112 [105;120]
Subgroup with obesity (n=8)	96.0 [88.5;104]	117 [104;130]
Asparagine		
Main group (n=16)	45.9 [41.5;50.3]	49.3 [45.4;53.2]
Subgroup without obesity (n=8)	50.2 [43.9;56.5]	46.4 [43.5;49.3]
Subgroup with obesity (n=8)	41.6 [36.7;46.5]	52.2 [45.2;59.2]
Aspartic Acid		
Main group (n=16)	12.6 [10;15.2]	15.6 [12.8;18.4]
Subgroup without obesity (n=8)	14.7 [10.5;18.9]	17.8 [13.4;22.2]
Subgroup with obesity (n=8)	10.4 [8;12.8]	13.5 [10.6;16.4]
Glutamine		
Main group (n=16)	716 [633;799]	778 [722;835]
Subgroup without obesity (n=8)	804 [739;867]	700 [649;751]
Subgroup with obesity (n=8)	628 [499;757]	856.[791;922]
Glutamic Acid		
Main group (n=16)	83.9 [61.3;107]	67.6 [56.5;78.7]
Subgroup without obesity (n=8)	71.1 [56.6;85.6]	75 [59.4;90.6]
Subgroup with obesity (n=8)	96.7 [54.2;139]	60.2 [45.2;75.2]
Glycine		
Main group (n=16)	221 [183;260]	249 [209;288]
Subgroup without obesity (n=8)	233 [189;276]	232 [204;261]
Subgroup with obesity (n=8)	210 [144;277]	266 [192;340]
Histidine		
Main group (n=16)	84.6 [78.1;91.1]	84.4 [77.2;91.6]
Subgroup without obesity (n=8)	79.4 [75.8;83]	77.9 [74.8;81]
Subgroup with obesity (n=8)	89.8 [78.1;102]	90.9 [77.9;104]
Isoleucine		
Main group (n=16)	68.8 [64.2;73.4]	60.4 [53;67.8]
Subgroup without obesity (n=8)	65.1 [59.3;70.9]	60 [49.4;70.6]
Subgroup with obesity (n=8)	72.4 [65.7;79.1]	60.9 [49.9;71.9]
Leucine		
Main group (n=16)	132 [117;148]	113 [101;126]
Subgroup without obesity (n=8)	127 [115;139]	119 [103;136]

Subgroup with obesity (n=8)	138 [110 - 166.]	108 [88.7 - 127]
Lysine		
Main group (n=16)	178 [169 - 187]	175 [165 - 186]
Subgroup without obesity (n=8)	173 [161 - 184]	166 [150 - 183]
Subgroup with obesity (n=8)	183 [169 - 196]	184 [173 - 195]
Methionine		
Main group (n=16)	24.7 [23.6 - 26.7]	24.4 [23.5 - 26.9]
Subgroup without obesity (n=8)	24 [22.5 - 25.4]	24.5 [21.9 - 26.8]
Subgroup with obesity (n=8)	25.4 [22.4 - 28.1]	24.4 [22 - 26.6]
Phenylalanine		
Main group (n=16)	63.1 [60.3 - 66.1]	61 [60.4 - 66]
Subgroup without obesity (n=8)	61.1 [57.9 - 64.1]	61.1 [57 - 64.9]
Subgroup with obesity (n=8)	65 [60.1 - 69.7]	61 [56.9 - 64.9]
Proline		
Main group (n=16)	183 [138 - 182]	205 [166 - 241]
Subgroup without obesity (n=8)	176 [137 - 209]	170 [140 - 196]
Subgroup with obesity (n=8)	190 [151 - 223]	240 [153 - 293]
Serine		
Main group (n=16)	109 [91.6 - 109]	116 [115 - 137]
Subgroup without obesity (n=8)	118 [106 - 130]	114 [102 - 125]
Subgroup with obesity (n=8)	98.8 [87.1 - 110]	117 [99.5 - 132]
Threonine		
Main group (n=16)	109 [97 - 121]	116 [106 - 127]
Subgroup without obesity (n=8)	118 [102 - 133]	112 [96.7 - 127]
Subgroup with obesity (n=8)	101 [83.1 - 118]	121 [105 - 136]
Tryptophan		
Main group (n=16)	59.6 [55.4 - 63.8]	61.5 [57 - 66]
Subgroup without obesity (n=8)	59.9 [54.9 - 64.9]	61.1 [53.4 - 68.8]
Subgroup with obesity (n=8)	59.3 [52.2 - 66.4]	61.9 [56.8 - 67]
Tyrosine		
Main group (n=16)	64.1 [58.7 - 69.5]	64 [58.2 - 69.8]
Subgroup without obesity (n=8)	60.2 [54.1 - 66.3]	60.2 [53.6 - 66.8]
Subgroup with obesity (n=8)	68 [59.6 - 76.4]	67.9 [58.7 - 77.1]
Valine		
Main group (n=16)	252 [231 - 273]	225 [206 - 245]
Subgroup without obesity (n=8)	230 [200 - 260]	221 [196 - 246]
Subgroup with obesity (n=8)	275 [256 - 294]	230 [198 - 261]

Table S1. Baseline fasting values of individual amino acid levels. Data are presented as means with 95% confidence intervals in brackets.

Table S2

**Uncorrected *P* values**							
	T2D vs CTRL	T2D,lean vs T2D,obese	T2D,lean vs CTRL,lean	T2D,lean vs CTRL,obese	T2D,obese Vs CTRL,lean	T2D,obese vs CTRL,obese	CTRL,obese vs CTRL,lean
Alanine	0.1438	0.0456	0.2530	0.7299	*0.0008*	0.0657	0.6554
Arginine	0.0266	0.3772	0.4062	0.2384	0.0104	0.5537	0.0324
Asparagine	0.3734	0.2942	0.0530	0.6905	0.1176	0.1558	0.1035
Aspartic Acid	0.0723	0.2168	0.1022	0.6424	0.0123	0.1347	0.2387
Glutamine	0.3543	0.0684	0.0322	0.2817	0.3284	*0.0024*	0.0379
Glutamic Acid	0.2038	0.6730	0.2833	0.3196	0.3627	0.2023	0.1315
Glycine	0.4066	0.9860	0.5888	0.4672	0.5631	0.4245	0.3603
Histidine	0.9563	0.3413	0.1199	0.1179	0.0741	0.0763	0.9112
Isoleucine	0.0637	0.3959	0.1272	0.5116	0.0735	0.9150	0.1081
Leucine	0.0200	0.4844	0.4913	0.1161	0.2787	0.3859	0.0126
Lysine	0.7412	0.6097	0.2891	0.1864	0.1566	0.1022	0.8883
Methionine	0.8726	0.7920	0.3818	0.7694	0.6491	0.9636	0.6891
Phenylalanine	0.3143	0.9982	0.2005	0.9848	0.2325	0.9879	0.1538
Proline	0.4054	0.6815	0.6190	0.3217	0.3911	0.2622	0.3274
Serine	0.3909	0.6612	0.0382	0.8895	0.0843	0.8301	0.1867
Threonine	0.4703	0.6048	0.1727	0.7930	0.3481	0.4526	0.2482
Tryptophan	0.4023	0.7555	0.8894	0.5913	0.7421	0.8625	0.3724
Tyrosine	0.9888	0.9863	0.1636	0.1911	0.1716	0.1982	0.9957
Valine	0.0855	0.7234	0.0264	0.9944	*0.0048*	0.6888	0.0314
Branch chained amino acids	0.0421	0.5950	0.0814	0.5350	0.0313	0.9550	0.0166
Total amino acids	0.8943	0.0260	0.6083	0.2462	0.3279	0.0274	0.3490
FDR-corrected *P* values							
	T2D vs CTRL	T2D,lean vs T2D,obese	T2D,lean vs CTRL,lean	T2D,lean vs CTRL,obese	T2D,obese Vs CTRL,lean	T2D,obese vs CTRL,obese	CTRL,obese vs CTRL,lean
Alanine	0.4916	0.3352	0.5903	0.8867	0.1176	0.4006	0.8526
Arginine	0.2977	0.6427	0.6427	0.5848	0.2977	0.7980	0.2977
Asparagine	0.6427	0.6268	0.3710	0.8530	0.4519	0.5004	0.4475
Aspartic Acid	0.4006	0.5591	0.4475	0.8507	0.2977	0.4715	0.5848
Glutamine	0.6427	0.4006	0.2977	0.6216	0.6427	0.1764	0.3120
Glutamic Acid	0.5350	0.8530	0.6216	0.6427	0.6427	0.5350	0.4715
Glycine	0.6427	0.9982	0.8223	0.7127	0.8036	0.6638	0.6427
Histidine	0.9982	0.6427	0.4519	0.4519	0.4006	0.4006	0.9890
Isoleucine	0.4006	0.6427	0.4675	0.7521	0.4006	0.9890	0.4519
Leucine	0.2977	0.7266	0.7295	0.4519	0.6216	0.6427	0.2977
Lysine	0.8869	0.8223	0.6250	0.5350	0.5004	0.4475	0.9811
Methionine	0.9811	0.9179	0.6427	0.9048	0.8519	0.9982	0.8530

Phenylalanine	0.6427	0.9982	0.5350	0.9982	0.5848	0.9982	0.5004
Proline	0.6427	0.8530	0.8272	0.6427	0.6427	0.6022	0.6427
Serine	0.6427	0.8526	0.3120	0.9811	0.4054	0.9533	0.5350
Threonine	0.7127	0.8223	0.5181	0.9179	0.6427	0.7003	0.5885
Tryptophan	0.6427	0.8956	0.9811	0.8223	0.8869	0.9811	0.6427
Tyrosine	0.9982	0.9982	0.5117	0.5350	0.5181	0.5350	0.9982
Valine	0.4054	0.8862	0.2977	0.9982	0.2352	0.8530	0.2977
Branch chained amino acids	0.3257	0.8223	0.4054	0.7787	0.2977	0.9982	0.2977
Total amino acids	0.9811	0.2977	0.8223	0.5885	0.6427	0.2977	0.6427

Table S2. Uncorrected and corrected *P* values from multiple comparisons of baseline fasting individual and total amino acids. CTRL: matched control group; Lean: Lean subgroups; obese: obese subgroups; T2D: Type 2 diabetes group; FDR: false discovery rate.

Table S3

	Type 2 diabetes group		Control group	
	Change from baseline (pmol/L)	Change from baseline (%)	Change from baseline (pmol/L)	Change from baseline (%)
Alanine				
Main group (n=16)	−58.9 [−78.7 - −39.1]	−14.2 [−19.4 - −9]	−44.3 [−58.5 - −30.1]	−12.4 [−16.5 - −8.3]
Subgroup without obesity (n=8)	−70.4 [−96.8 - −44]	−17.9 [−25.2 - −10.6]	−46.1 [−71 - −21.2]	−14.1 [−21.3 - −6.9]
Subgroup with obesity (n=8)	−47.4 [−76.5 - −18.3]	−10.5 [−17.2 - −3.8]	−42.6 [−57.9 - −27.3]	−10.8 [−14.9 - −6.7]
Arginine				
Main group (n=16)	−5.7 [−9.5 - −1.9]	−5.6 [−9.4 - −1.8]	−7.8 [−11.3 - −4.3]	−7.2 [−10.5 - −3.9]
Subgroup without obesity (n=8)	−5.5 [−12 - 1]	−5 [−11.4 - 1.4]	−6.3 [−10.5 - −2.1]	−5.8 [−9.5 - −2.1]
Subgroup with obesity (n=8)	−5.9 [−10.3 - −1.5]	−6.3 [−11 - −1.6]	−9.2 [−15 - −3.4]	−8.7 [−14.3 - −3.1]
Asparagine				
Main group (n=16)	−5.7 [−7.6 - −3.8]	−12.1 [−15.9 - −8.3]	−6.3 [−7.7 - −4.9]	−12.8 [−15.2 - −10.4]
Subgroup without obesity (n=8)	−7.3 [−9.8 - −4.8]	−14.5 [−19.5 - −9.5]	−6 [−7.7 - −4.3]	−12.9 [−16.3 - −9.5]
Subgroup with obesity (n=8)	−4.1 [−6.5 - −1.7]	−9.7 [−15.1 - −4.3]	−6.7 [−8.9 - −4.5]	−12.6 [−16.3 - −8.9]
Aspartic Acid				
Main group (n=16)	−1.9 [−3.3 - −0.5]	−9.8 [−20.9 - 1.3]	−1.2 [−2.5 - 0.1]	−5.3 [−13.2 - 2.6]
Subgroup without obesity (n=8)	−2.8 [−5.2 - −0.4]	−12.9 [−32.6 - 6.8]	−2.7 [−4.1 - −1.3]	−14.3 [−20.8 - −7.8]
Subgroup with obesity (n=8)	−0.9 [−2.2 - 0.4]	−6.6 [−18.1 - 4.9]	0.2 [−1.6 - 2]	3.7 [−8.1 - 15.5]
Glutamine				
Main group (n=16)	−35.4 [−50.7 - −20.1]	−4.7 [−6.8 - −2.6]	−49.6 [−68.7 - −30.5]	−6.1 [−8.6 - −3.6]
Subgroup without obesity (n=8)	−44.7 [−64.5 - −24.9]	−5.5 [−7.7 - −3.3]	−38.8 [−67.2 - −10.4]	−5.2 [−9.1 - −1.3]
Subgroup with obesity (n=8)	−26.1 [−48.7 - −3.5]	−4 [−7.6 - −0.4]	−60.4 [−85.6 - −35.2]	−7.1 [−10.2 - −4]
Glutamic Acid				
Main group (n=16)	−5.7 [−12.1 - 0.7]	−4.3 [−16.3 − 4.2]	−6.2 [−13.2 - 0.8]	−6.6 [−18.1 − 1.7]
Subgroup without obesity (n=8)	−4.4 [−12.2 - 3.4]	−3.7 [−16.2 − 7.6]	−13.4 [−21.2 - −5.6]	−15.9 [−23.9 − −8.4]
Subgroup with obesity (n=8)	−7 [−17.6 - 3.6]	−5 [−23.9 − 10.8]	1 [−8.7 - 10.7]	2.6 [−16.8 − 19.6]
Glycine				
Main group (n=16)	−26.2 [−35.5 - −16.9]	−11.5 [−15 - −8]	−28.9 [−36.3 - −21.5]	−11.4 [−13.7 - −9.1]
Subgroup without obesity (n=8)	−29.1 [−41.1 - −17.1]	−12.2 [−16.7 - −7.7]	−29.2 [−40 - −18.4]	−12.1 [−15.7 - −8.5]
Subgroup with obesity (n=8)	−23.2 [−38 - −8.4]	−10.7 [−16.4 - −5]	−28.6 [−39.4 - −17.8]	−10.6 [−13.7 - −7.5]
Histidine				
Main group (n=16)	−0.7 [−7.4 - 6]	−0.8 [−8.8 - 7.2]	−4.4 [−6.5 - −2.3]	−5.5 [−8.1 - −2.9]
Subgroup without obesity (n=8)	−5.7 [−8.1 - −3.3]	−7.1 [−10 - −4.2]	−5 [−8.4 - −1.6]	−6.3 [−10.5 - −2.1]
Subgroup with obesity (n=8)	4.2 [−8.5 - 16.9]	5.5 [−9.5 - 20.5]	−3.9 [−6.6 - −1.2]	−4.7 [−8.1 - −1.3]
Isoleucine				
Main group (n=16)	−7.5 [−9.8 - −5.2]	−11 [−14.6 - −7.4]	−9.4 [−11.6 - −7.2]	−15.5 [−18.7 - −12.3]
Subgroup without obesity (n=8)	−8.5 [−12.1 - −4.9]	−13.2 [−19.2 - −7.2]	−9.2 [−11.7 - −6.7]	−15.3 [−18.5 - −12.1]
Subgroup with obesity (n=8)	−6.6 [−9.4 - −3.8]	−8.8 [−12.3 - −5.3]	−9.5 [−13.4 - −5.6]	−15.7 [−21.6 - −9.8]
Leucine				
Main group (n=16)	−17 [−27.9 - −6.1]	−11.4 [−20.3 − −4.6]	−18.4 [−28.5 - −8.3]	−15.2 [−24.6 - −7.8]

Subgroup without obesity (n=8)	−12.1 [−18.9 - −5.3]	−10 [−15.6 - −4.6]	−16.6 [−20.9 - −12.3]	−14 [−17.1 - −11]
Subgroup with obesity (n=8)	−21.9 [−42.8 - −1]	−12.8 [−28.7 - 0.9]	−20.3 [−40.7 - 0.1]	−16.4 [−34 - −0.8]
Lysine				
Main group (n=16)	−11.3 [−15.8 - −6.8]	−6.4 [−8.9 - −3.9]	−11.7 [−17.2 - −6.2]	−6.8 [−9.9 - −3.7]
Subgroup without obesity (n=8)	−13.7 [−20.8 - −6.6]	−7.6 [−11.4 - −3.8]	−12.7 [−19.2 - −6.2]	−7.9 [−11.8 - −4]
Subgroup with obesity (n=8)	−9 [−14.4 - −3.6]	−5.1 [−8.3 - −1.9]	−10.8 [−19.9 - −1.7]	−5.7 [−10.5 - −0.9]
Methionine				
Main group (n=16)	−2.9 [−3.8 - −2]	−11.9 [−15.3 - −8.5]	−2.8 [−3.7 - −1.9]	−11.2 [−14.7 - −7.7]
Subgroup without obesity (n=8)	−3.4 [−4.9 - −1.9]	−13.8 [−19.6 - −8]	−3.1 [−4.5 - −1.7]	−12.2 [−17.1 - −7.3]
Subgroup with obesity (n=8)	−2.5 [−3.3 - −1.7]	−10 [−13.4 - −6.6]	−2.5 [−3.7 - −1.3]	−10.2 [−15.3 - −5.1]
Phenylalanine				
Main group (n=16)	−7.2 [−8.6 - −5.8]	−11.6 [−14.1 - −9.1]	−7.6 [−9 - −6.2]	−12.5 [−14.9 - −10.1]
Subgroup without obesity (n=8)	−8.8 [−10.7 - −6.9]	−14.5 [−17.8 - −11.2]	−8.4 [−10.2 - −6.6]	−13.7 [−16.2 - −11.2]
Subgroup with obesity (n=8)	−5.5 [−6.8 - −4.2]	−8.7 [−11.1 - −6.3]	−6.8 [−9.1 - −4.5]	−11.3 [−15.3 - −7.3]
Proline				
Main group (n=16)	−16.1 [−20.7 - −11.5]	−8.9 [−11 - −6.8]	−19.2 [−24.2 - −14.2]	−10.1 [−12.3 - −7.9]
Subgroup without obesity (n=8)	−16.5 [−20.5 - −12.5]	−9.9 [−13 - −6.8]	−18.6 [−23.1 - −14.1]	−11.4 [−14.5 - −8.3]
Subgroup with obesity (n=8)	−15.7 [−24.4 - −7]	−7.8 [−10.5 - −5.1]	−19.7 [−28.9 - −10.5]	−8.8 [−11.7 - −5.9]
Serine				
Main group (n=16)	−9.8 [−13.1 - −6.5]	−8.6 [−11.6 - −5.6]	−9.9 [−13.3 - −6.5]	−8.3 [−10.9 - −5.7]
Subgroup without obesity (n=8)	−12.8 [−16.3 - −9.3]	−11 [−14.2 - −7.8]	−11.3 [−15 - −7.6]	−10.1 [−13.7 - −6.5]
Subgroup with obesity (n=8)	−6.7 [−11.7 - −1.7]	−6.2 [−10.8 - −1.6]	−8.6 [−14.5 - −2.7]	−6.6 [−10.1 - −3.1]
Threonine				
Main group (n=16)	−9.5 [−12.8 - −6.2]	−8.2 [−10.4 - −6]	−13.3 [−16 - −10.6]	−11.4 [−13.5 - −9.3]
Subgroup without obesity (n=8)	−10.4 [−13.8 - −7]	−8.6 [−11 - −6.2]	−12.6 [−16.7 - −8.5]	−11.1 [−14.2 - −8]
Subgroup with obesity (n=8)	−8.5 [−14.4 - −2.6]	−7.8 [−11.8 - −3.8]	−13.9 [−17.5 - −10.3]	−11.7 [−14.8 - −8.6]
Tryptophan				
Main group (n=16)	−5.3 [−7.4 - −3.2]	−9.1 [−12.6 - −5.6]	−3.1 [−4.8 - −1.4]	−4.8 [−7.7 - −1.9]
Subgroup without obesity (n=8)	−6.1 [−9.3 - −2.9]	−10.4 [−16.1 - −4.7]	−2.8 [−5.8 - 0.2]	−3.8 [−8.6 - 1]
Subgroup with obesity (n=8)	−4.5 [−7.2 - −1.8]	−7.8 [−12 - −3.6]	−3.5 [−5.5 - −1.5]	−5.8 [−9.4 - −2.2]
Tyrosine				
Main group (n=16)	−8.2 [−9.7 - −6.7]	−13 [−15.5 - −10.5]	−9.2 [−11.1 - −7.3]	−14.2 [−16.9 - −11.5]
Subgroup without obesity (n=8)	−9.1 [−11.6 - −6.6]	−15.2 [−19.3 - −11.1]	−8.6 [−11.9 - −5.3]	−13.9 [−18.8 - −9]
Subgroup with obesity (n=8)	−7.3 [−8.8 - −5.8]	−10.9 [−13.2 - −8.6]	−9.7 [−11.5 - −7.9]	−14.4 [−16.9 - −11.9]
Valine				
Main group (n=16)	−10.9 [−14.6 - −7.2]	−4.6 [−6.3 - −2.9]	−18.1 [−23.6 - −12.6]	−8.4 [−10.9 - −5.9]
Subgroup without obesity (n=8)	−14.1 [−19.2 - −9]	−6.4 [−8.9 - −3.9]	−16.2 [−22.3 - −10.1]	−7.8 [−11 - −4.6]
Subgroup with obesity (n=8)	−7.8 [−12.6 - −3]	−2.8 [−4.5 - −1.1]	−20.1 [−29.6 - −10.6]	−9.1 [−13.1 - −5.1]

Table S3. Absolute and relative change from baseline in individual amino acids. Data are presented as means with 95% confidence intervals in parentheses.

Table S4

Uncorrected *P* values							
	T2D vs CTRL	T2D,lean vs T2D,Obese	T2D,Lean vs CTRL,Lean	T2D,Lean vs CTRL,obese	T2D,obese Vs CTRL,lean	T2D,obese vs CTRL,obese	CTRL,obese vs CTRL,lean
Alanine	0.3482	0.1659	0.1180	0.4848	0.4444	0.9497	0.3482
Arginine	0.8153	0.7638	0.4125	0.8872	0.4195	0.5582	0.8153
Asparagine	0.6324	0.2245	0.5687	0.3420	0.9117	0.5098	0.6324
Aspartic Acid	0.8997	0.5915	0.1756	0.2710	0.0199	0.2545	0.8997
Glutamine	0.8921	0.5136	0.4209	0.6818	0.4634	0.3238	0.8921
Glutamic Acid	0.1257	0.9156	0.5682	0.3349	0.0723	0.5483	0.1257
Glycine	0.9683	0.6909	0.5684	0.6872	0.5353	0.9741	0.9683
Histidine	0.7475	0.1303	0.3188	0.1613	0.5786	0.1936	0.7475
Isoleucine	0.5709	0.2355	0.5723	0.0171	0.9068	0.0547	0.5709
Leucine	0.2984	0.7318	0.4249	0.8858	0.7533	0.7639	0.2984
Lysine	0.9089	0.3451	0.5534	0.3105	0.5094	0.8709	0.9089
Methionine	0.6448	0.2937	0.3788	0.4800	0.5790	0.9721	0.6448
Phenylalanine	0.7091	0.0139	0.2444	0.0124	0.3378	0.3172	0.7091
Proline	0.4303	0.3403	0.6152	0.1099	0.2490	0.7016	0.4303
Serine	0.6870	0.1142	0.0858	0.2152	0.1905	0.8847	0.6870
Threonine	0.0731	0.7341	0.1499	0.2206	0.8001	0.2683	0.0731
Tryptophan	0.2255	0.4824	0.1958	0.2426	0.5335	0.5258	0.2255
Tyrosine	0.7344	0.0979	0.7707	0.2947	0.8606	0.1666	0.7344
Valine	0.5973	0.0316	0.2948	0.0159	0.6253	0.0142	0.5973
Branch chained amino acids	0.4584	0.6105	0.3026	0.1897	0.6266	0.1705	0.4584
Total amino acids	0.9788	0.2657	0.6833	0.2274	0.6427	0.4717	0.9788
FDR-corrected *P* values							
	T2D vs CTRL	T2D,lean vs T2D,Obese	T2D,Lean vs CTRL,Lean	T2D,Lean vs CTRL,obese	T2D,obese Vs CTRL,lean	T2D,obese vs CTRL,obese	CTRL,obese vs CTRL,lean
Alanine	0.8693	0.8391	0.8391	0.8391	0.8693	0.8693	0.9788
Arginine	0.9129	0.9363	0.9129	0.8693	0.9546	0.8693	0.8693
Asparagine	0.9546	0.8770	0.8391	0.8693	0.8391	0.9546	0.8693
Aspartic Acid	0.9788	0.9546	0.8693	0.8391	0.8391	0.4876	0.8391
Glutamine	0.9261	0.9546	0.8693	0.8693	0.8988	0.8693	0.8391
Glutamic Acid	0.8693	0.8391	0.9546	0.8693	0.8391	0.8391	0.8693
Glycine	0.8391	0.9788	0.8988	0.8693	0.8988	0.8693	0.9788
Histidine	0.8391	0.9129	0.8391	0.8391	0.8391	0.8693	0.8391
Isoleucine	0.8693	0.8693	0.8391	0.8693	0.4876	0.9546	0.8391
Leucine	0.8693	0.8391	0.9129	0.8693	0.9546	0.9129	0.9129
Lysine	0.8693	0.9546	0.8391	0.8693	0.8391	0.8693	0.9546

Methionine	0.8693	0.8776	0.8391	0.8693	0.8693	0.8693	0.9788
Phenylalanine	0.6028	0.9064	0.4876	0.8391	0.4876	0.8391	0.8391
Proline	0.9129	0.8693	0.8391	0.8770	0.8391	0.8391	0.9047
Serine	0.8391	0.8988	0.8391	0.8391	0.8391	0.8391	0.9546
Threonine	0.8693	0.8391	0.9129	0.8391	0.8391	0.9261	0.8391
Tryptophan	0.8391	0.8391	0.8693	0.8391	0.8391	0.8693	0.8693
Tyrosine	0.8693	0.9129	0.8391	0.9129	0.8391	0.9546	0.8391
Valine	0.9507	0.8693	0.6028	0.8391	0.4876	0.8770	0.4876
Branch-chained amino acids	0.8391	0.8693	0.8770	0.8391	0.8391	0.8770	0.8391
Total amino acids	0.8693	0.9788	0.8391	0.8988	0.8391	0.8776	0.8693

Table S4. Uncorrected and corrected *P* values from multiple comparisons of relative change from baseline individual and total amino acids. CTRL: matched control group; Lean: Lean subgroups; obese: obese subgroups; T2D: Type 2 diabetes group FDR; false discovery rate.
